# Macronutrient manipulations of cheese resulted in lower energy content without compromising its satiating capacity

**DOI:** 10.1017/jns.2017.73

**Published:** 2018-02-05

**Authors:** Thea Toft Hansen, Anders Sjödin, Christian Ritz, Simon Bonnet, Sanne Kellebjerg Korndal

**Affiliations:** 1 Department of Nutrition, Exercise and Sports, Section for Obesity Research, Faculty of Science, University of Copenhagen, Rolighedsvej 26, DK-1958 Frederiksberg C, Denmark; 2 Department of Nutrition, Bel Group (Europe and North America), Paris, France

**Keywords:** Appetite, Satiety, Satiation, Cheese, Accumulated energy intake, Appetite sensations, Appetite quotient, AQ, appetite quotient, HP/HF, high-protein/high-fat cheese, HP/LF, high-protein/low-fat cheese, LP/HF, low-protein/high-fat cheese, SQ, satiety quotient, VAS, visual analogue scale

## Abstract

Manipulation of food's macronutrient composition in order to reduce energy content without compromising satiating capacity may be helpful in body weight control. For cheeses, substituting fat with protein may provide such opportunity. We aimed at examining the acute effect of cheeses with different macronutrient compositions on accumulated energy intake and subjective appetite sensation. A total of thirty-nine normal-weight (average BMI 24·4 kg/m^2^) men and women completed the partly double-blind, randomised crossover study with high-protein/low-fat (HP/LF, 696 kJ), high-protein/high-fat (HP/HF, 976 kJ) and low-protein/high-fat (LP/HF, 771 kJ) cheeses. After overnight fasting, 80 g cheese were served with 70 g bread, 132 g juice and 125 g coffee/tea/water. *Ad libitum* spaghetti bolognaise was served after 3 h and energy intake assessed. Subjective appetite ratings were assessed using visual analogue scales. Composite appetite scores were calculated and evaluated relatively to energy intake. Total accumulated energy intake was 188·3 (se 97·4) kJ lower when consuming the HP/LF compared with the HP/HF (*P* ≤ 0·05), but, compared with the LP/HF cheese, the difference was not significant (177·0 (se 100·4) kJ lower; *P* = 0·08). In relation to energy intake, the composite appetite score was lower when consuming the HP/LF compared with the HP/HF (*P* = 0·003) and the LP/HF (*P* = 0·007) cheeses. Thereby, no compensatory eating following consumption of the HP/LF compared with the HP/HF cheese was found. The HP/LF cheese resulted in an increased feeling of satiety in relation to its lower energy content compared with both HP/HF and LP/HF cheeses.

## Introduction

Satiety is the feeling which develops at the end of an eating episode and promotes inhibition over further eating as well as between meals^(^^1^^)^. Promotion of satiety between meals is favourable in the light of the growing obesity burden. Feelings of hunger have been reported as a major reason for failed weight-loss success^(^^2^^,^^3^^)^; thus increasing the feeling of satiety holds a potential for body weight management. The feeling of being satiated and not able to eat more is subjective and hence impossible to measure directly. Feelings of appetite are usually assessed by visual analogue scales (VAS), on which people mark their feeling of, for example, hunger, satiety, fullness and desire to eat^(^^4^^,^^5^^)^.

Much research has been performed in order to understand the satiating effects of the different macronutrients, and many studies have pointed towards protein being the macronutrient most competent of enhancing satiety^(^^6^^–^^8^^)^. One study also found a dose-dependent effect of a higher protein content leading to higher ratings of postprandial fullness and lower ratings of hunger^(^^9^^)^. Furthermore, high-protein diets have been shown to have a positive effect on weight loss^(^^10^^)^ and weight-loss maintenance^(^^8^^,^^11^^)^.

In addition to the satiating properties of nutrients, it is of interest to investigate the satiating capacity of foods relative to their energy content by comparing the effects of equal amounts and thus study the satiating power relative to the energy content^(^^12^^,^^13^^)^. To investigate this, the satiety quotient (SQ) can be used as a measure of the extent to which the food eaten during the eating episode reduces subjective appetite per unit of energy^(^^1^^)^. The SQ takes into account the pre-meal appetite sensations and considers the energy content of the meal and has been shown to be associated with the following energy intake^(^^14^^)^. For meals differing in energy content, the SQ is thereby considered a more valid indicator of satiety than the 1 h postprandial AUC^(^^14^^,^^15^^)^. In addition, the SQ has been shown to have a good reliability when assessed under controlled conditions^(^^14^^)^. In view of the present obesity burden, increasing the feeling of satiety for as little energy as possible might be a potential approach. If similar levels of satiety can be obtained despite a lower energy intake, this could be a powerful tool for weight control. Hence, it is of relevance to examine if foods differing in energy content are able to produce the same degree of satiety.

The main objective of the present study was to examine whether cheeses with different protein, fat and thereby energy content affect energy intake at a following *ad libitum* meal assessing the accumulated energy intake. The secondary objective was to examine postprandial effects on subjective appetite sensations in relation to the products’ energy content. The objectives were investigated based on the following hypotheses analysed according to this prioritisation:

1. Macronutrient manipulation resulting in a cheese with high protein content as well as low fat content and thereby a lower energy content will have a higher satiating capacity in relation to its energy content, resulting in lower accumulated energy intake, compared with a cheese with similar protein content but higher fat content and thereby a higher energy content.
2. Macronutrient manipulation resulting in a cheese with low protein content as well as high fat content and thereby a lower energy content will have a lower satiating capacity in relation to its energy content, resulting in similar accumulated energy intake, compared with a cheese with similar fat content but higher protein content and thereby a higher energy content.
3. Macronutrient manipulation resulting in cheeses with low energy content but varying protein and fat content will affect the satiating capacity of the cheeses differently resulting in lower accumulated energy intake when the protein content is high and the fat content is low compared with when the protein content is low and the fat content is high.

## Materials and methods

### Subjects, inclusion and exclusion criteria, ethics

Based on an online advertisement, seventy men and women were recruited for the study. After telephone and in-person screenings, forty healthy men and women aged between 18 and 60 years were found eligible. Further inclusion criteria were BMI of 20·0–31·9 kg/m^2^, regular consumption of breakfast (≥4 times weekly) and regular menstrual periods for the women (for practical reasons, women were not tested in the same phase of their menstrual period at all study visits). Smoking, self-reported weight changes (±3 kg) in the previous 3 months and use of medication affecting appetite and/or body weight were exclusion criteria. The in-person screening included measurement of non-fasting body weight to the nearest 0·1 kg on a calibrated scale (Lindell Tronic 8000; Samhall Lavi) wearing light clothing and having emptied the bladder. Height without shoes was measured to the nearest 0·5 cm using a wall-mounted stadiometer (Seca). Self-reported dislike, allergy towards or unwillingness to consume study products served as part of the experimental procedures resulted in screening failure.

The study was reported to the local ethical committee (journal no. 15012763), but due to the design of the study not involving any biological outcomes, ethical approval of the study was not required. The study protocol and study forms complied with the relevant sections of the Declaration of Helsinki. Before any study-related procedures, all participants signed written informed consent after written and oral information and were assigned individual three-digit identification codes. Participant recruitment and testing took place at the Department of Nutrition, Exercise and Sports, University of Copenhagen, Denmark between September and November 2015. The study is registered on www.clinicaltrials.gov (NCT02582723).

### Study procedures

A partly double-blind, randomised crossover design with three experimental conditions was employed; high-protein/low-fat (HP/LF) hard cheese, high-protein/high-fat (HP/HF) hard cheese and low-protein/high-fat (LP/HF) cream cheese. All cheeses were already accessible on the European market. Visual difference between the hard cheeses (HP/HF and HP/LF) and the cream cheese (LP/HF) was impossible to blind for the study participants during eating occasions. The experimental condition was concealed for the investigator during serving of the food; the food tray was covered during serving and not uncovered by the participant until the investigator had left the room. No visual differences were apparent between the hard cheeses (HP/LF and HP/HF) and the study participants did not receive any information about the different contents of cheeses during the study. Order of the conditions was randomised using simple block randomisation stratified on sex and BMI (≤25·9 and ≥26·0 kg/m^2^) and the test days were carried out at least 4 d apart using the same experimental procedures. For standardisation, no alcohol or intense physical activity was allowed 48 h before the procedures, and the participants arrived at the study facilities in the morning after an overnight fast (from 22.00 hours) using non-strenuous means of transportation. Fasting body weight while wearing light clothing and having emptied the bladder was measured to the nearest 0·1 kg on a calibrated scale (Lindell Tronic 8000; Samhall Lavi) before study-related procedures were performed at each test day. Participants were settled one or two into rooms of approximately 12 m^2^. When there were two participants at the same time, the room was separated in two by a removable wall and the meals was served simultaneously.

Participants were served a standardised breakfast meal including 80 g of the experimental product, 70 g white bread, 132 g orange juice and 125 g coffee/tea/water (free of choice but repeated for all three conditions and milk and sugar were not allowed) of which they had to consume the entire amount (Table 1). The amounts of bread and juice were determined in advance of the study in order for the breakfast containing the HP/HF hard cheese to meet 2000 kJ. Thereby, the amount of bread and juice with the HP/HF hard cheese was used as the reference and these amounts for bread and juice were kept constant regardless of the condition (i.e. resulting in different energy content of the breakfast meals dependent on the condition). The reference breakfast meal was determined aiming at an energy content of the meal of 2000 kJ, which corresponds to approximately 20 % of the daily energy requirements for an average adult^(^^16^^)^. The protein source in the three cheeses is derived from cows’ milk proteins; however, the distribution of protein types varies slightly between the hard cheeses (both approximately 95 % casein and 5 % whey protein) and the cream cheese (approximately 90 % casein and 10 % whey protein).

**Table 1.** Nutritional values of the breakfast meals: energy and macronutrient content and energy density of the test products and total of the breakfast meal

	HP/LF	HP/HF	LP/HF	Bread	Juice	Total
Energy (kJ)	696			748	277	1721
		976				2000
			771			1796
Amount (g)	80			70	132	282
		80				282
			80			282
Protein (g)	20			4·9	0·1	25
		17·2				22
			4·4			9
Carbohydrate (g)	Traces			35·2	16·1	51
		Traces				51
			3·2			55
Fat (g)	9·6			1·4	0	11
		18·4				20
			17·2			19
Energy density (kJ/100 g)	870			1069	210	2149
		1220				2499
			964			2243

HP/LF, high-protein/low-fat cheese; HP/HF, high-protein/high-fat cheese; LP/HF, low-protein/high-fat cheese.

At 3 h post-breakfast, a homogeneous spaghetti bolognese *ad libitum* meal (8000 kJ, 15 % energy (E%) as protein, 30 E% as fat and 55 E% as carbohydrate) was served for the assessment of energy intake. A total of 250 g water was served along with the *ad libitum* meal during the first condition, and the amount consumed was free of choice but repeated for the following conditions. Total accumulated energy intake was calculated from the energy intake at the breakfast meal and the *ad libitum* meal and used as the primary indication of appetite.

Before and after the meal and at 30-min intervals, subjective appetite ratings were assessed using VAS on a tablet (Lenovo® thinkpad® tablet 10) with special designed software for electronic VAS (Acqui version 1; Jakob Lund Laugesen, University of Copenhagen). On the screen, questions for perceptions of satiety, fullness, hunger, desire to eat and predicted prospective food consumption (for the detailed questions asked, see the Supplementary material) appeared one by one in a random order together with a 100 mm horizontal unbroken line with words anchored at each end describing the extremes. Participants responded to the question by placing a finger on the horizontal line corresponding to their perceived feeling at that particular time resulting in a small vertical line. This could be moved to either end of the horizontal line until continuation to the next question. Change in answers upon continuation was not possible and responded questions were no longer visible. This method has previously been validated and found to be similar to pen-and-paper VAS^(^^17^^,^^18^^)^. Subjective appetite ratings were used as a secondary assessment of appetite. Additionally, VAS assessing subjective evaluations of the cheese products (liking, taste and texture) were applied after consuming the breakfast meals.

During the 3 h interval between meals, sedentary activities (studying, reading, etc.) not involving appetite-stimulating issues were allowed but the participants were not allowed to leave the study facility nor consume any foods or drinks. The 3-h interval between the meals was found to be optimal in order for the participants to be hungry enough to eat again but not so hungry that the satiating effect from the breakfast meals disappeared and thereby blurred the opportunity to see potential differences in the satiating capacity^(^^19^^)^. Furthermore, a 3-h interval between breakfast and the next meal corresponds well with the general Danish meal pattern.

### Sample size

The study was designed to have a 90 % power to detect a difference of 400 kJ in *ad libitum* energy intake between the conditions with thirty-five subjects completing. The sample size calculation was based on the assumption that the within-subject standard deviation in *ad libitum* energy intake was 500 kJ^(^^20^^)^.

### Statistical analyses

The primary outcome was the accumulated energy intake from the breakfast (including the cheeses) and the energy intake from the *ad libitum* meal. Secondary outcomes of the study were the accumulated food intake from the breakfast (including the cheeses) and the energy and food intake from the *ad libitum* meal as well as the VAS assessed throughout the test days used to calculate the appetite quotients (AQ) (inspired by SQ; see equation below)^(^^21^^)^, relating appetite sensations assessed by VAS to the energy intake. Additionally, palatability evaluations of the cheeses were applied using VAS assessing liking, taste and texture of the cheeses.

For energy and food intake (energy (kJ) and weight (g)) as well as palatability evaluations linear mixed models were fitted, including adjustment for visit and subject (the latter included as a random effect). In an additional subgroup analysis for women only, total accumulated energy and food intake were also adjusted for phase of menstrual cycle. According to the prioritisation of the hypotheses outlined previously, the comparisons of interest (HP/LF *v.* HP/HF; LP/HF *v.* HP/HF; HP/LF *v.* LP/HF) were estimated from these models. No adjustment for multiplicity was applied.

Composite appetite scores were calculated for each time point that the appetite ratings were assessed according to the formula:
1
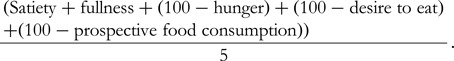

AQ based on the composite appetite scores were calculated for each time point that appetite ratings were assessed inspired by the previously validated equation^(^^21^^)^:
2
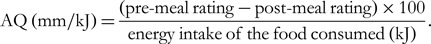

The AQ expresses the satiating capacity of the food, and the lower the AQ, the more satiating effect per unit of energy intake of the food consumed.

To analyse differences in the AQ relative to the energy consumed (kJ), linear mixed models were used. Specifically, condition time interactions were evaluated. The models included adjustment for visit and subject (the latter included as a random effect). Based on these models, pairwise comparisons between conditions were obtained according to the prioritisation of the hypotheses. Again no adjustment for multiplicity was applied.

For all models, assumptions of normality and homogeneity of variance were assessed through visual inspection of quantile–quantile plots and plots of residual against the fitted values.

Statistical analyses were carried out using Stata/IC 11.2 (StataCorp) and are presented as means with standard deviations or standard errors as appropriate. Statistical significance was declared using a significance level of 0·05.

## Results

### Study population

A total of forty participants were included in the study. One participant dropped out and refused further contact before any study procedures were carried out.

Baseline characteristics are presented in Table 2 showing the overall characteristics of completed subjects and separated between sexes.

**Table 2.** Subject characteristics at baseline (Mean values and standard deviations; ranges; numbers of subjects)

	Overall (*n* 39)		Female (*n* 28)		Male (*n* 11)	
	Mean	sd	Mean	sd	Mean	sd
Age (years)	26·3	10·9	25·4	11·5	28·6	9·3
Range	20·3–55·9		20·3–55·9		20·9–52·0	
Height (cm)	171·4	8·2	167·5	5·1	181·1	6·1
Weight (kg)*	71·5	9·4	69·4	10·1	76·9	4·2
BMI (kg/m^2^)†	24·4	3·1	23·7	3·3	26·3	1·4
Education‡						
About 2 years academic, vocational or less	5		2		3	
3–4·5 years academic	8		7		1	
>5 years academic	26		19		7	
Civil status						
Single or divorced	12		9		3	
Married or relationship	27		19		8	

* Fasting measurement obtained at first condition (no difference between conditions).

† Based on non-fasting body weight measured at screening.

‡ Ongoing or highest completed.

### Energy intake

All participants consumed the entire amount of breakfast served. Total accumulated energy (i.e. fixed breakfast + *ad libitum* meal) intake was 188·3 (se 97·4) kJ lower when consuming the HP/LF compared with the HP/HF cheese (*P* ≤ 0·05) (Table 3). Furthermore, total accumulated food intake was 34·9 (se 17·9) g higher when consuming LP/HF compared with HP/HF cheese (*P* ≤ 0·05). Also, a tendency (*P* = 0·08) for a 177·0 (se 100·4) kJ lower total accumulated energy intake when consuming the HP/LF compared with the LP/HF cheese was observed.

**Table 3.** Total accumulated energy/food (i.e. fixed breakfast + *ad libitum* test meal) intake and energy/food intake from the *ad libitum* test meal (Mean values with their standard errors)

	HP/LF		HP/HF		LP/HF		P*		
	Mean	se	Mean	se	Mean	se	HP/LF *v.* HP/HF	LP/HF *v.* HP/HF	HP/LF *v.* LP/HF
Total accumulated energy intake (kJ)	4191	162	4381	160	4384	156	0·05	0·91	0·08
Total accumulated food intake (g)	727	29	711	29	749	28	0·35	0·05	0·31
*Ad libitum* test meal energy intake (kJ)	2470	162	2380	160	2588	152	0·35	0·05	0·31
*Ad libitum* test meal food intake (g)	445	29	429	29	467	28	0·35	0·05	0·31

HP/LF, high-protein/low-fat cheese; HP/HF, high-protein/high-fat cheese; LP/HF, low-protein/high-fat cheese.

* Differences were analysed by linear mixed model.

No differences were found in *ad libitum* energy or food intake (both *P* = 0·35) after consuming the HP/LF compared with the HP/HF cheese, indicating no compensation. A compensation was indicated by a 193·8 (se 99·5) kJ higher a*d libitum* energy intake and a 34·9 (se 17·9) g higher food intake when consuming the LP/HF compared with the HP/HF cheese (both *P* ≤ 0·05) (Table 3).

For women, total accumulated energy and food intake were adjusted for phase of menstrual cycle, but this did not affect the results (data not shown, *P* = 0·39 and *P* = 0·88, respectively).

### Appetite quotient

**Fig. 1.**
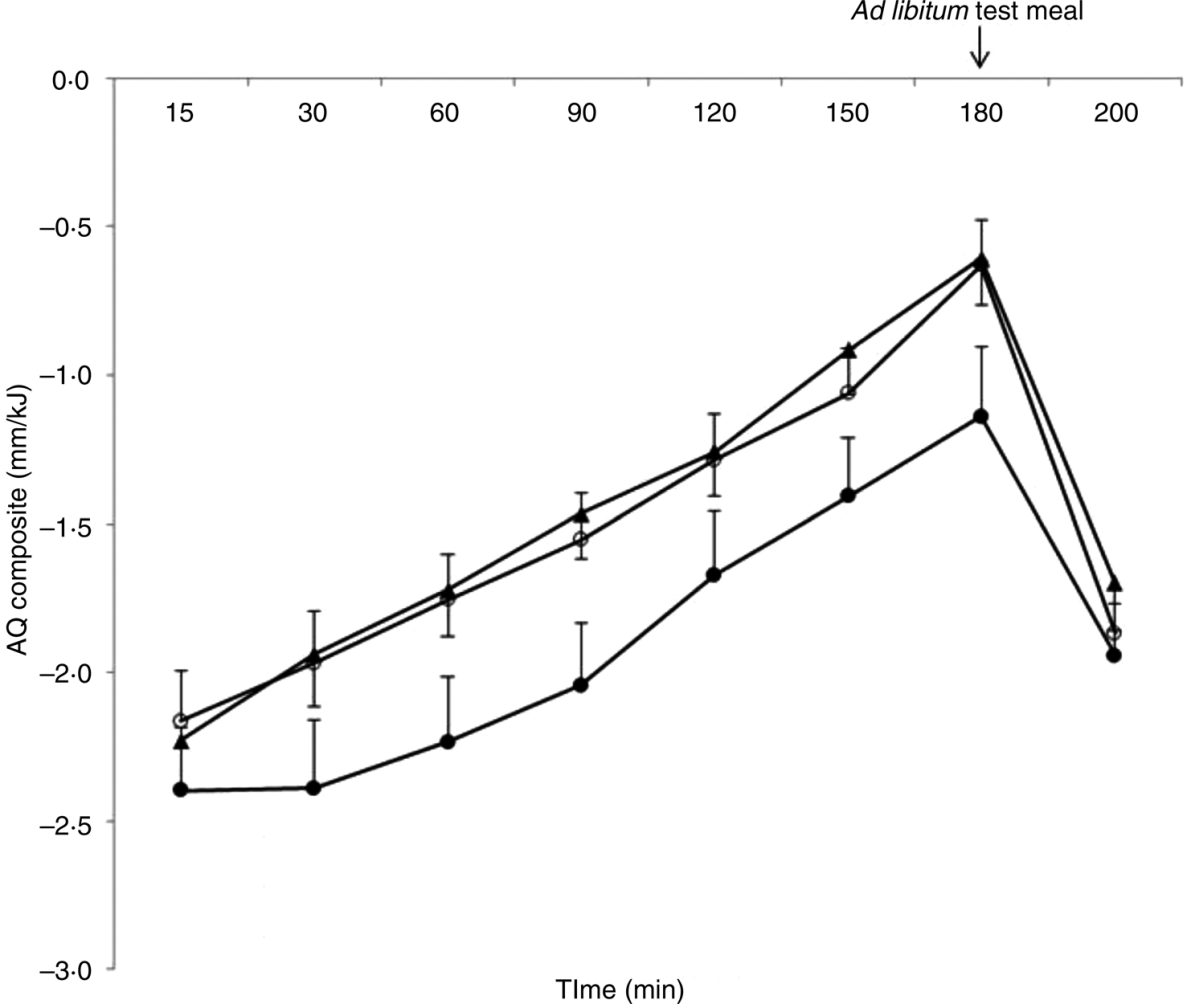
Appetite quotients (AQ) (see equation 2) for the composite score (see equation 1) of appetite sensations assessed by visual analogue scales pre- and 15–180 min post-breakfast as well as pre and post the *ad libitum* test meal (180–200 min) (mm/kJ). Values are means, with standard errors represented by vertical bars. Differences were analysed by linear mixed model. No interaction between condition and time for the AQ for the composite appetite score was found (*χ*^2^ = 7·73 (df = 14), *P* = 0·90). For the main effect of condition, the average AQ for the composite appetite score was lower when consuming the high-protein/low-fat cheese (HP/LF; –●–) compared with the high-protein/high-fat cheese (HP/HF; –○– ) (*P* = 0·003) and the low-protein/high-fat cheese (LP/HF; –▲–) (*P* = 0·007). No difference between the HP/HF and the LP/HF cheeses was found (*P* = 0·82).

For the AQ for the composite appetite score, there was no interaction between condition and time (*χ*^2^ = 7·73 (df = 14), *P* = 0·90). For the main effect of condition, the average AQ for the composite appetite score was lower when consuming the HP/LF compared with the HP/HF (*P* = 0·003) and the LP/HF (*P* = 0·007) cheeses, showing an increased satiating effect per unit of energy of the HP/LF cheese independent of time compared with both conditions (Fig. 1).

There was no difference between the HP/HF and the LP/HF cheeses (*P* = 0·82).

### Cheese palatability

No differences were found in the palatability evaluations of the cheese products (all *P* ≥ 0·11) (Table 4).

**Table 4.** Palatability evaluations of the cheese products assessed by visual analogue scales (Mean values with their standard errors)

	HP/LF		HP/HF		LP/HF		P*		
	Mean	se	Mean	se	Mean	se	HP/LF *v.* HP/HF	LP/HF *v.* HP/HF	HP/LF *v.* LP/HF
Liking†	58·6	3·6	55·4	3·5	56·3	3·4	0·32	0·88	0·27
Taste‡	54·1	3·6	52·6	3·1	52·9	3·4	0·67	0·91	0·60
Texture‡	53·3	3·8	48·2	3·8	49·5	4·7	0·18	0·78	0·11

HP/LF, high-protein/low-fat cheese; HP/HF, high-protein/high-fat cheese; LP/HF low-protein/high-fat cheese.

* Differences were analysed by linear mixed model.

† 0, not at all; 100, extremely much.

‡ 0, not good at all; 100 extremely good.

## Discussion

In the present study, lower total accumulated energy intake after consumption of the HP/LF compared with the HP/HF cheese was found. For the *ad libitum* meal 3 h following the breakfast meal, we found no difference in energy or food intake between the HP/LF cheese and any of the two other conditions. Energy and food intake were higher when consuming the LP/HF compared with the HP/HF cheese. These findings suggest no compensation in *ad libitum* energy or food intake following the HP/LF cheese despite this breakfast meal providing less energy. In contrast, compensatory eating was indicated following consumption of the LP/HF cheese, which also contained less energy compared with the HP/HF cheese. These results indicate that the protein content of a meal may determine the food eaten at the next meal to a greater extent than the energy content. In correspondence, Leidy *et al.* showed that both normal-protein and high-protein breakfasts increased daily fullness compared with skipping breakfast; however, only the high-protein breakfast reduced evening snacking and also increased daily fullness more compared with the normal-protein breakfast^(^^22^^)^. These findings are in line with the present study showing a larger effect on appetite of the HP/LF cheese per unit of energy consumed. This indicates an increased feeling of satiety for less energy compared with the HP/HF and the LP/HF cheeses. As obesity is a major health problem globally, maintaining the satiating capacities of foods despite providing less energy might provide tools for weight management when included as part of a diet. Feelings of hunger have been found to be a risk factor for unsuccessful weight loss^(^^23^^)^ and obese individuals have previously reported increased feelings of hunger following energy restriction^(^^24^^)^, illustrating the importance of appetite sensations in weight management.

The absence of compensatory eating following the HP/LF compared with the HP/HF cheese might be due to the high protein content in both type of cheese, as a higher protein content has been shown to result in increased feelings of satiety^(^^9^^)^. The amount of protein in the breakfast meals comprising the HP/LF and HP/HF cheeses was almost similar (25·0 and 22·2 g, respectively); whereas the protein content of the breakfast meal containing the LP/HF cheese was considerably lower (9·4 g). Thus, the high-protein cheeses may provide equal satiating effects due to the nearly similar protein content. This may explain why no difference in energy or food intake was found at the *ad libitum* meal between the high protein conditions. This is also consistent with previous studies showing no difference in *ad libitum* energy intake despite clear dose-dependent increases in satiety and fullness with increased protein contents in isoenergetic meals^(^^7^^,^^9^^)^. This is supported by the AQ across the test day, which showed a significantly larger effect on appetite of the HP/LF cheese per unit of energy consumed. The HP/LF cheese provided lower feelings of appetite across the test day compared with the HP/HF cheese despite less energy (1720 and 2000 kJ, respectively) and compared with the LP/HF cheese, which contained closer to equal amounts of energy (1720 and 1796 kJ, respectively) but less protein (25·0 and 9·4 g, respectively). These results indicate that decreased feelings of appetite following a meal can be obtained for less energy by higher protein content of these study products, which corresponds to previous findings of enhanced satiety from products and meals high in protein^(^^6^^,^^8^^,^^9^^,^^22^^,^^25^^)^. This is further supported by the results on energy intake at the *ad libitum* meal following consumption of the LP/HF cheese, showing increased energy and food intake. Thus, compensatory eating was indicated when the meal contained less energy and protein, but not when the meal only contained less energy. This illustrates that the difference in energy intake from the breakfast meals due to the different energy content of the test products does not solely explain the difference found in total accumulated energy intake. If the different energy content of the test product alone caused the difference in total accumulated energy intake, the total accumulated energy intake after consuming the LP/HF cheese should have been lower, due to the low energy content of the LP/HF cheese. As this is close to similar to the total accumulated energy intake after consuming the HP/HF cheese, this supports the hypothesis that higher protein intake makes the difference.

### Strength and limitations of the study

It has previously been shown that adults tend to regulate energy intake over a 24 h period^(^^26^^)^. In order to fully exclude compensatory eating following consumption of the different cheeses, a full assessment of energy intake over an extended period would be relevant. Moreover, demonstration of a sustained effect on energy intake and appetite is needed to result in any effect on body weight. Fixed amounts of the test products were served with the fixed breakfast foods, which resulted in breakfast meals differing in energy density and texture of the test products. Energy density, texture and palatability of the foods may affect appetite. Thereby this addresses a limitation of the study, since it was not possible to avoid differences in these parameters between conditions in order to study the accumulated energy intake and the satiating capacity of the cheeses relative to their energy content. Furthermore, the type of the protein may affect the appetite differently. However, the hard cheeses only varied slightly from the cream cheese, with the cream cheese having the highest content of whey protein, thereby potentially increasing the satiating effect of the cream cheese compared with the hard cheeses^(^^27^^)^. The amount of test product was decided based on having sufficient amount of protein, i.e. resulting in 80 g cheese. This is a relatively high cheese consumption, which limits the generalisability of the results. Furthermore, no data on, for example, appetite-related hormones were obtained. Belza *et al.*^(^^9^^)^ previously showed a dose-dependent effect of protein on glucagon-like peptide-1 (GLP-1) and peptide YY (PYY) 3–36. It would have been interesting to study if the differences in protein content between the conditions resulted in different responses in, for example, GLP-1, PYY or ghrelin. Furthermore, despite adjusting the results for menstrual cycle phase, it may be a limitation of the study that the included women were not tested within the same phase of their menstrual cycle.

The fixed amounts of the study products served make it possible to investigate the effects on appetite of equal amount of foods even with varying energy content. People tend to eat a constant amount of food in weight independent of the energy content^(^^28^^)^, making it interesting to investigate effects on appetite of equal amount of foods with different energy contents. In the present study, we actually found lower appetite ratings for less energy after consuming equal amounts of foods despite different energy content. The sensations of appetite may be based on learned satiating effects of foods, which probably determine the amount of food needed to feel satiated to a greater extent than the energy content of the food^(^^29^^)^. To study the effect of different protein and energy content on appetite, it is thereby an advantage to examine the total accumulated energy and food intake by serving a fixed amount of the meal including the study product rather than focusing on these meals being isoenergetic.

### Conclusion

Despite less energy from the breakfast comprising the HP/LF cheese, no compensatory eating was found at the following meal, resulting in lower total accumulated energy intake when consuming the HP/LF compared with the HP/HF cheese. Similar total accumulated energy intake was observed after the breakfast comprising the LP/HF and HP/HF cheeses. Thus, less energy from the breakfast comprising the LP/HF cheese was somewhat compensated for at the following meal when consuming the LP/HF cheese but not the HP/LF cheese. The HP/LF cheese furthermore resulted in an increased feeling of satiety for less energy compared with both the HP/HF and the LP/HF cheeses. However, the increased feeling of satiety from the HP/LF cheese only tended to result in lower accumulated energy intake compared with the LP/HF cheese. Thus, it can thereby be concluded that cheese with a high protein content enhances satiety regardless of fat content and thereby provides the potential for decreased energy intake when included as part of a diet.

## Supplementary material

For supplementary material accompanying this paper visit https://doi.org/10.1017/jns.2017.73. click here to view supplementary material
