# Supplementary material for: Macronutrient manipulations of cheese resulted in lower energy content without compromising its satiating capacity
Source: J Nutr Sci. 2018 Feb 5;7:e7. doi: 10.1017/jns.2017.73 (PMC5799611; doi:10.1017/jns.2017.73)
Supplement: Supplementary file 1 [file S2048679017000738sup001.doc]

**Supplementary material**

Supplementary Table S1. Visual analogue scale questions used to assess subjective appetite

| I am completely empty | How satisfied do you feel? | I cannot eat another bite |
| --- | --- | --- |
|  |
|  |
| Not at all full | How full do you feel? | Totally full |
|  |
|  |
| I am not hungry at all | How hungry do you feel? | I have never been more hungry |
|  |
| Not at all strong | How strong is your desire to eat? | Totally strong |
|  |
|  |
| Nothing at all | How much do you think you can eat? | A lot |
|  |
|  |  |  |
